# Molecular docking analysis of stachydrine and sakuranetin with IL-6 and TNF-α in the context of inflammation

**DOI:** 10.6026/97320630017363

**Published:** 2021-02-28

**Authors:** Lavanya Prathap, Selvaraj Jayaraman, Anitha Roy, Preetha Santhakumar, M Jeevitha

**Affiliations:** 1 Department of Anatomy, Saveetha Dental College and Hospitals, Saveetha Institute of Medical and Technical Sciences, Chennai - 600 077, Tamil Nadu, India; 2 Department of Biochemistry, Saveetha Dental College and Hospitals, Saveetha Institute of Medical and Technical Sciences, Chennai - 600 077, Tamil Nadu, India; 3 Department of Pharmacology, Saveetha Dental College and Hospitals, Saveetha Institute of Medical and Technical Sciences, Chennai - 600 077, Tamil Nadu, India; 4 Department of Physiology, Saveetha Dental College and Hospitals, Saveetha Institute of Medical and Technical Sciences, Chennai - 600 077, Tamil Nadu, India; 5 Department of Periodontics, Saveetha Dental College and Hospitals, Saveetha Institute of Medical and Technical Sciences, Chennai - 600 077, Tamil Nadu, India

**Keywords:** IL-6, TNF-α, inflammation, molecular docking

## Abstract

Inflammation is a process triggered by pro-inflammatory cytokines and anti-inflammatory molecules. Therefore, it is of interest to document the anti-inflammatory activity of Stachydrine and Sakuranetin against the inflammatory target proteins IL-6 and TNF-α by using molecular docking analysis. Both compounds showed good binding features with the selected target proteins. Compared to Sakuranetin, the Stachydrine have low binding energy and good hydrogen bond interactions. Hence, data show that Stachydrine possessed high and specific inhibitory activity on tumor necrosis factor-α and interleukin-6.

## Background

Inflammation, including fatty liver diseases such as alcoholic liver disease (ALD) and non-alcoholic fatty liver disease (NAFLD), is an integral component of both all-acute and chronic liver disorder. In recent decades, it has been convincingly shown that immune mediators, especially pro-inflammatory cytokines, have been able to regulate many of the main characteristics of liver diseases, including acute liver failure, acute phase response, steatosis, cholestasis, hypergammaglobulinemia, and production of fibrosis [1,2]. Cytokines function to control the human immune response in combination with various cytokine inhibitors and soluble cytokine receptors. There is a growing understanding of their physiological role in inflammation and their pathological role in systemic inflammatory conditions. Interleukin (IL)-1 receptor antagonists, IL-4, IL-10, IL-11, and IL-13 are major anti-inflammatory cytokines. Under different conditions, leukaemia inhibitory factor, interferon-alpha, IL-6, and transforming growth factor (TGF)-β are known as either anti-inflammatory or proinflammatory cytokines. IL-1, TNF-alpha, and IL-18 unique cytokine receptors also act as proinflammatory cytokine inhibitors [3]. Cytokine, IL-6, is involved not only in the response to inflammation and infection, but also in the modulation of metabolic, regenerative, and neural processes. An interleukin that has pro- and anti-inflammatory effects is IL-6. It points out that classic signalling regulates interleukin-6 regenerative or anti-inflammatory actions, whereas interleukin-6 pro-inflammatory responses are more mediated by trans-signaling. This is relevant because the neutralising anti-interleukin-6 receptor monoclonal antibody tocilizumab has recently been licenced for therapeutic blockade of interleukin-6 in the therapy of inflammatory diseases [4]. TNF-α is a cytokine that is active in systemic inflammation and is a member of the acute inflammation inducing cytokine family. Different kinds of cells in the body contain TNF-alpha. Kupffer cells in the liver generate TNF-alpha predominantly, and TNF-alpha is also an essential mediator in different physiological processes, such as inflammation, cell proliferation and apoptosis [5,6]. Efficient treatment of inflammatory autoimmune disorders such as rheumatoid arthritis [7] is given by blocking pro-inflammatory cytokines. However other chronic autoimmune disorders, such as systemic lupus erythematosus (SLE) [8], have a therapeutic benefit by blocking pro-inflammatory cytokines [8]. Therefore, it is of interest to report the molecular docking analysis of stachydrine and sakuranetin with IL-6 and TNF-α in the context of inflammation.

## Methodology

### Protein structure:

The X-ray crystallography structures of IL-6(1ALU), TNF-α (1TNF) were downloaded from RCSB PDB (https://www.rcsb.org/) [9]. The retrieved complexes has water molecules, ions and in some their respective ligands. These complexes were prepared by deleting water molecules, ions, ligands and then it was saved as .pdb format.

### Ligand structures:

The three dimensional (3D) structure of sakuranetin and stachydrine was retrieved in .sdf format from PubChem (https://pubchem.ncbi.nlm.nih.gov/). The .sdf format of compounds was converted to .pdb using the Online Smiles Translator. 

### Molecular Docking:

AutoDock Vina v1.1 [10,11] programmes have been used to investigate the binding of receptor ligands. Using AutoDockTools v1.5.6 (The Scripps Research Institute, La Jolla, CA, USA), the required input files for AutoDock Vina were prepared. File preparation involved changing the type of atom, removing molecules of water, and adding atoms of polar hydrogen and Gasteiger charges. With the default grid spacing, the grid box size was retained. The structure files were saved in the format PDBQT was used for molecular docking analysis. The study was conducted using AutoDock Vina v1.1 (Scripps Research Institute). For analysis, the outcomes with the best conformation and energy were selected. Using Pymol [12], The docked structures were visualized using Pymol [12].

## Results and Discussion:

**Figure 1:**
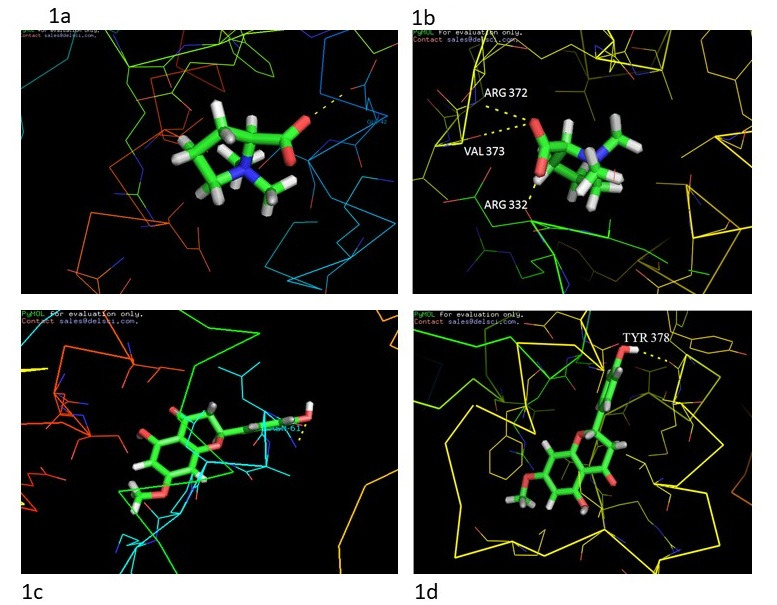
Molecular docking interactions data: (a) IL-6 with Stachydrine; b) TNF-α with Stachydrine c) IL-6 with Sakuranetin d) TNF-α with Sakuranetin

Molecular docking means that the drug is correctly absorbed and binds with the receptor. The lowest docking energy is the most significant interaction between ligand and receptor. Autodock4.2 is used to evaluate affinity, binding conformation, best ligand and target orientation. For both ligands, out of the multiple docking poses, only those that had the highest docking score were selected. The binding force, the number of hydrogen bond interactions and the amino acids participating in the interactions have been seen in Table 1 for IL-6 and TNF-α. Docking tests of IL-6 compounds showed that stachydrine has the lowest binding energy of −5.9 kcal/mol. With IL-6, Stachydrine formed one hydrogen bond with GLU-4 2 (Figure 1a). Stachydrine's binding by TNF-alpha inhibition, demonstrating a strong binding energy of -6.8 kcal/mol and hydrogen bonding with ARG-332, ARG-372, VAL-373 is shown (Figure 1b). Figure 1c shows the binding of IL-6-Sakuranetin, resulting in a transition in the conformation of IL-6 protein by ligand by creating a signal by binding to the site of the targeted IL-6 protein. The figure shows the ligand-receptor interactions of the yellow dotted line with the residue ASN-61 and shows the binding energy of-5.8 kcal/mol. This indicates the high affinity of Sakuranetin to IL-6. The hydrogen bond association between residue interactions of Sakuranetin present in the TNF-alpha protein cavity was represented in Figure 1d. Hydrogen bond interaction with TYR-378 residue depicted by a yellow dotted line is seen in the diagram. Thus it is understood that TNF-a has a particular goal as the primary pathway that plays a significant role based on the molecular docking of different target proteins associated with the inflammatory cascade in liver diseases. The activated monocytes/macrophages and T-cells develop tumour necrosis factor alpha and are located on the macrophage cell membrane when it is not activated. When macrophages are activated, their release depends on TACE, which is the disintegrin- and metalloprotease-containing enzyme (ADAM) responsible for processing the inactive form of tumour necrosis factor alpha into its active soluble form that binds to TNF-alpha such that receptor signalling pathways for apoptosis, inflammatory response or development of cytokines are formed. Inhibition of the release of soluble TNF-alpha can thus, inhibit the inflammatory cascade in patients.

## Conclusion

We document the molecular docking based binding features of stachydrine and sakuranetin with (interleukin-6) IL-6 and (tumor necrosis factor-α) TNF-α in the context of inflammation for further consideration. Data shows that stachydrine have optimal binding features with the tumor necrosis factor-α and interleukin-6.

## Figures and Tables

**Table 1:** Molecular docking results of stachydrine and sakuranetin

S.No	Protein Name	Binding Energy kcal/mol	Hydrogen bond interaction
Stachydrine			
1	IL-6	-5.9	GLU-42
2	TNF-α	-6.8	ARG-332
			ARG-372
			VAL-373
Sakuranetin			
1	IL-6	-5.8	ASN-61
2	TNF-α	-6.4	TYR-378
